# Nutritional Risk in Patients Admitted to Medical Oncology Departments: Prevalence, Associated Factors and Clinical Outcomes in a Multicentre Prospective Study

**DOI:** 10.3390/nu18081296

**Published:** 2026-04-20

**Authors:** Francisco Javier Teigell Muñoz, Laia Llobera Rius, Laura Medina Ortega, Pablo Villacé Gallego, Javier Marco-Hernández, Esther Paula Fernández Fernández, Anna Esquerrà Molas, Leyre Liesa Delgado, Luis Manuel Sáez Urán, Jesús González Olmedo, Carlos Heredia-Mena, Javier Romero Hernández, Mariona Llaberia-Torrelles, María Jesús Delgado Heredia, Nagore Bazaga Rodas, Lidia Fernández-Cordón, Cristina Macía-Rodríguez, Jade Soldado, Adrián García-Villa

**Affiliations:** 1 Co-Management and Consultative Medicine Working Group, Spanish Society of Internal Medicine (SEMI), 28008 Madrid, Spain; 2 Department of Internal Medicine, Hospital Universitario Infanta Cristina, IDIPHISA, 28981 Madrid, Spain; 3 Catalan Institute of Oncology (ICO), Hospital Universitari Germans Trias i Pujol, 08916 Badalona, Spain; 4 Consorci Corporació Sanitària Parc Taulí, 08208 Sabadell, Spain; 5 Medical Oncology Department, Hospital de Barcelona, 08025 Barcelona, Spain; 6 Department of Internal Medicine, Hospital Clínic de Barcelona, 08036 Barcelona, Spain; 7 Hospital de Mataró, 08304 Barcelona, Spain; 8 Medical Oncology Department, Hospital Universitario Arnau de Vilanova, 25198 Lleida, Spain; 9 Hospital Universitario Virgen de las Nieves, 18014 Granada, Spain; 10 Department of Internal Medicine, Hospital Universitario 12 de Octubre, 28041 Madrid, Spain; 11 Medical Oncology Department, Hospital San Pedro, 26006 Logroño, Spain; 12 Hospital del Mar, 08003 Barcelona, Spain; 13 Department of Internal Medicine, Hospital Universitario La Princesa, 28006 Madrid, Spain; 14 Hospital Quirónsalud A Coruña, 15008 A Coruña, Spain; 15 Department of Internal Medicine, Hospital Virgen del Puerto, 10600 Plasencia, Spain

**Keywords:** nutritional risk, malnutrition screening tool (MST), cancer, hospitalised patients, medical oncology, clinical outcomes

## Abstract

**Background/Objectives**: Malnutrition and nutritional vulnerability are common in patients with cancer and are associated with adverse clinical outcomes, particularly among hospitalised patients. However, data specifically describing nutritional risk in patients admitted to medical oncology departments remain limited. This study aimed to estimate the prevalence of nutritional risk at hospital admission and to evaluate factors associated with nutritional risk and its clinical consequences. **Methods**: The REGIO registry is a national multicentre prospective cohort including adult patients with solid tumours admitted to medical oncology departments in 17 Spanish hospitals between February 2024 and January 2025. Nutritional risk was assessed within the first working day of hospitalisation using the Malnutrition Screening Tool (MST), with a score ≥ 2 indicating nutritional risk. Multivariable logistic regression models were used to identify factors associated with nutritional risk and its association with prolonged hospital stay and mortality. **Results**: A total of 1229 patients were included (median age 67.8 years; 59% male; 64% metastatic disease). Nutritional risk was identified in 53% of patients. In multivariable analysis, poorer functional status, tumour progression, recent exposure to cytotoxic chemotherapy, and tumour types with higher nutritional impact were independently associated with nutritional risk. Patients at nutritional risk had longer hospital stays (median 10 vs. 7 days; *p* < 0.001), a higher likelihood of prolonged hospitalisation (adjusted OR 1.38), and increased mortality at 30 days (adjusted OR 1.63) and 60 days after discharge (adjusted OR 1.53). **Conclusions**: In this large multicentre cohort, nutritional risk was highly prevalent and independently associated with worse clinical outcomes, supporting the clinical relevance of systematic nutritional screening at hospital admission in patients with cancer.

## 1. Introduction

Malnutrition is a clinical condition characterised by an imbalance in tissue metabolism and nutritional reserves, and is associated with functional impairment and poorer clinical outcomes [1,2,3]. In the context of illness, disease-related malnutrition (DRM) is defined by the presence of systemic inflammation together with insufficient nutrient intake or impaired nutrient utilisation, as seen in patients with cancer [4].

DRM is particularly prevalent among patients with cancer and has been associated with poorer prognosis and reduced survival. Cancer increases the risk of malnutrition through several mechanisms, including reduced dietary intake due to anorexia and treatment-related gastrointestinal toxicity, increased energy and protein requirements associated with chronic systemic inflammation, and reduced anabolic stimuli such as physical activity [4].

The negative impact of disease-related malnutrition (DRM) among patients with cancer has been widely documented, affecting survival, quality of life, and healthcare costs [4]. Indeed, it is estimated that 10–20% of deaths among patients with cancer are attributable to malnutrition rather than to the tumour itself [5]. In hospitalised patients with cancer, DRM has also been associated with a higher risk of complications, poorer tolerance to anticancer treatments, reduced survival, and longer hospital stays, with consequent functional, clinical, and economic consequences [4,6]. Despite this impact, nutritional screening at hospital admission, recommended by major clinical guidelines [7,8,9,10], remains underutilised in hospitalised oncology patients, thereby limiting early identification of nutritional risk and timely access to appropriate nutritional interventions [4,11,12].

The prevalence of disease-related malnutrition (DRM) in patients with cancer has been widely studied, although reported estimates vary widely. Although prevalence is generally high, reported estimates range from 20% to 80% [6,7,13,14], with recent meta-analyses reporting pooled prevalences around 40% in patients with cancer [15]. This wide variability may be influenced by several factors, including the country in which the study was conducted, the healthcare setting (outpatient versus hospitalised patients), the timing of the nutritional assessment, the type of malignancy, tumour stage and oncological status (controlled versus progressive disease), and the nutritional assessment tool used. Specifically, in hospitalised patients with cancer, the reported prevalence of DRM ranges from 19% to 89% in previous studies [6,11,16,17,18,19].

In Spain, two major multicentre cohorts have reported the prevalence of disease-related malnutrition (DRM) in hospitalised patients with cancer: a subanalysis of the PREDyCES cohort [16] and a subgroup analysis of the SeDREno study [17], reporting prevalences of 33.4% and 29.7%, respectively. However, these studies have two main limitations. First, they were not specifically designed to evaluate patients with cancer, which resulted in a smaller oncology-specific sample size compared with other international cohorts [14]. Second, they included patients hospitalised across different hospital departments and for heterogeneous reasons, which may limit their applicability to the clinical context of medical oncology units. As a result, data specifically addressing nutritional risk in patients admitted to medical oncology departments remain scarce.

Therefore, the primary objective of this study was to estimate the prevalence of nutritional risk at hospital admission among patients with cancer admitted to medical oncology departments in Spain. As secondary objectives, we analysed the factors associated with nutritional risk and its impact on length of hospital stay, mortality, and hospital readmission.

## 2. Materials and Methods

### 2.1. Study Design and Participants

The REGIO registry is a national multicentre, prospective, observational, and longitudinal study promoted by the Spanish Society of Internal Medicine (SEMI), involving 17 centres and 38 investigators. Adult patients with solid tumours requiring hospital admission for any reason to the medical oncology departments of the participating centres were included (Supplementary Table S1). Patients admitted exclusively for diagnostic procedures, planned administration of antineoplastic treatment (i.e., scheduled inpatient admissions for treatment delivery, such as multi-day intravenous therapies), or comfort care in the context of imminent end of life were excluded. In addition, patients receiving systemic treatment in outpatient settings (e.g., day hospital or infusion units with same-day discharge) were not included, as they do not require hospital admission. Patients undergoing cancer-related surgical procedures were also not included, as in the Spanish healthcare system, they are routinely managed and hospitalised under surgical specialties rather than in medical oncology departments. Patients who declined to provide written informed consent were also excluded.

The recruitment period spanned from 1 February 2024 to 31 January 2025. Patients were selected using systematic sampling based on the time of hospital admission, including the first patient whose electronic admission record was registered after 17:00 on the previous day. To minimise interference with routine clinical activity and to reduce potential seasonal selection bias, a maximum of seven patients per month per investigator were included over twelve consecutive months.

Demographic variables, medical history, functional status, oncological history, and hospital size were recorded. Comorbidity was assessed using the non-cancer Charlson Comorbidity Index, derived from the original index [20], with oncological variables excluded due to their redundancy. Variables related to hospital admission (reason for admission, length of stay, and discharge destination) were also collected, and patients were followed clinically for up to 60 days after discharge.

### 2.2. Assessment of Nutritional Risk

Nutritional risk was assessed using the Malnutrition Screening Tool (MST) during the first working day of hospitalisation, with a score of ≥2 considered indicative of nutritional risk, which corresponds to the validated and widely accepted cut-off for this tool. The MST is a simple screening tool based on recent unintentional weight loss and decreased appetite, with higher scores reflecting greater nutritional risk (Supplementary Table S1). It is a screening tool validated in hospitalised oncology patients [4,21], with a reported sensitivity of 48–79%, specificity of 83–94%, and an area under the curve (AUC) ranging from 0.70 to 0.83 [21,22,23,24]. Given the multicentre design of the registry and its broad clinical scope, a simple and standardised screening tool was considered the most feasible approach for systematic assessment at hospital admission. Accordingly, the aim of this study was to identify nutritional risk rather than to establish a formal diagnosis of malnutrition. MST also offers several practical advantages, including its simplicity, rapid administration, and low interobserver variability [7,25]. Weight and height were recorded at admission, and body mass index (BMI) was calculated. Low BMI was defined, according to GLIM (Global Leadership Initiative on Malnutrition) criteria [2], as BMI < 20 kg/m^2^ in patients aged < 70 years or <22 kg/m^2^ in those aged ≥ 70 years.

### 2.3. Statistical Analysis

Quantitative variables were described as mean ± standard deviation (SD) or median (interquartile range [IQR]), according to their distribution, which was assessed using the Shapiro–Wilk test. Categorical variables were expressed as absolute frequencies and percentages. Comparisons between groups were performed using Student’s *t*-test or Welch’s *t*-test for normally distributed quantitative variables, and the Mann–Whitney U test otherwise. Categorical variables were compared using the χ^2^ test or Fisher’s exact test, as appropriate.

To identify factors associated with nutritional risk at hospital admission and to evaluate its clinical consequences (prolonged hospital stay, in-hospital mortality, and mortality 30 and 60 days after discharge), multivariable logistic regression models were used. Variable selection was based on clinical and epidemiological criteria and on their association in the univariable analysis, prioritising model parsimony. Potential collinearity between variables was also assessed.

Missing data were evaluated for all variables included in the analyses. The proportion of missing values was low for most variables (<5%). Multivariable analyses were therefore performed using complete-case datasets including patients with available data for all variables included in each model. Given the low proportion of missing data, imputation procedures were not considered necessary.

In the multivariable models, only tumour types with a frequency ≥ 1.5% in the cohort were included in order to avoid unstable estimates in underrepresented categories. Tumours were grouped a priori into three predefined clinical categories according to their nutritional impact, as described in previous studies [11,14,16,18]. In particular, tumours affecting the upper gastrointestinal tract, head and neck, and pancreas were classified as high impact due to their well-established association with reduced oral intake, malabsorption, and cancer cachexia, whereas breast cancer and melanoma were considered low impact, with intermediate categories including the remaining tumour types. 

Length of hospital stay was analysed both as a continuous variable and as a dichotomous variable. Prolonged hospitalisation was defined as a length of stay above the 75th percentile of the cohort, a commonly used approach in hospital outcome studies to identify patients with extended hospitalisation. Survival was estimated using Kaplan–Meier curves, which were compared using the log-rank test. Sensitivity analyses were performed by categorising relevant continuous variables, such as age and the non-cancer Charlson Comorbidity Index, in order to assess the robustness of the results. Results were expressed as odds ratios (ORs) with 95% confidence intervals (95% CIs). A *p*-value < 0.05 was considered statistically significant.

All analyses were performed using Jamovi software (version 2.6.44).

## 3. Results

### 3.1. Patient Characteristics

A total of 1229 patients were included; the median age was 67.8 years (IQR 15.75), and 59.0% were male. At admission, 64.3% of patients had metastatic disease and 38.8% had a baseline ECOG performance status ≥ 2 (Table 1). The proportion of missing data was low for most variables and is detailed in Supplementary Table S3.

The most frequent malignancies were lung cancer (29.0%), colorectal cancer (11.8%), breast cancer (9.4%), and pancreatic cancer (7.7%). At admission, 65.4% of patients had uncontrolled oncological disease, defined as either recent diagnosis or disease progression. Regarding prior oncological treatments, 59.2% of patients had received cytotoxic chemotherapy within the eight weeks preceding admission, 20.3% immunotherapy, 13.3% targeted molecular therapies, and 3.4% hormonal therapy.

The median length of hospital stay was 8 days (IQR 10). In-hospital mortality was 9.2%, reaching 27.6% at 60 days after discharge, and the overall 60-day readmission rate was 33.4% (Table 1).

### 3.2. Prevalence of Nutritional Risk and Associated Risk Factors

Overall, 53.0% of patients (n = 641) were identified as being at nutritional risk according to MST. In addition, 19.4% of patients had a low BMI according to GLIM criteria. Patients at nutritional risk had a lower mean body weight (66.7 ± 14.9 kg vs. 72.0 ± 14.5 kg; *p* < 0.001) and a significantly lower mean BMI (24.1 ± 4.9 vs. 26.2 ± 4.8; *p* < 0.001) compared with those without nutritional risk. Despite this, 72.7% of patients classified as being at nutritional risk had a BMI within the normal range, highlighting the limitations of BMI as a standalone marker of nutritional status.

The prevalence of nutritional risk was higher in men than in women (57.2% vs. 47.1%; *p* < 0.001), and also increased with worse functional status, with a prevalence of 31.9% in patients with ECOG 0–1 compared with 67.0% in those with ECOG 3–4 (*p* < 0.001). Regarding tumour type, the highest prevalences of nutritional risk were observed in oesophageal (73.0%), gastric (68.6%), pancreatic (71.0%) and head and neck cancers (57.7%), whereas the lowest prevalences were found in breast cancer (33.9%) and melanoma (33.1%) (Table 2). Nutritional risk was also more frequent in patients with uncontrolled oncological disease, both at initial diagnosis (60.8%) and in those with disease progression (57.5%), compared with patients with stable disease (43.5%), partial response (48.5%), or complete response (30.7%). No significant differences were observed according to metastatic status (54.3% vs. 51.0%; *p* = 0.27) (Table 3).

In the univariate analysis (Table 4), the male sex was associated with a higher likelihood of being at nutritional risk (OR 1.50; 95% CI: 1.19–1.89). Poorer functional status was also associated with a higher likelihood of nutritional risk (ECOG 2 vs. 0–1: OR 2.47; 95% CI: 1.89–3.27; ECOG 3–4 vs. 0–1: OR 2.84; 95% CI: 1.94–4.16). Significant associations were also observed with tumour progression, tumours with high nutritional risk and recent treatment with cytotoxic chemotherapy. No statistically significant associations were identified for age, non-tumour Charlson Comorbidity Index, metastatic disease or hospital type.

Multivariable analysis was performed in 1035 patients (Table 4). In the adjusted model, functional status remained independently associated with nutritional risk, with a higher likelihood observed in patients with ECOG 2 compared with ECOG 0–1 (OR 2.78; 95% CI: 1.91–4.62). Clinical status at admission, defined as tumour progression versus stable disease, was independently associated with a higher likelihood of being at nutritional risk (OR 1.88; 95% CI: 1.43–2.47). Recent treatment with cytotoxic chemotherapy also remained independently associated with nutritional risk (OR 1.41; 95% CI: 1.07–1.84). Regarding tumour type, an independent association with nutritional risk was observed, with a higher likelihood in tumours with intermediate nutritional impact (OR 1.82; 95% CI: 1.16–2.84), particularly in those with high nutritional impact (OR 3.32; 95% CI: 1.97–5.60), compared with tumours with low impact. Neither age (entered as a continuous variable) nor the non-tumour Charlson Comorbidity Index showed an independent association in the main model. The association observed for male sex in the univariate analysis did not remain significant after multivariable adjustment. In the sensitivity analyses, when age was categorised, only patients aged 80 years or older showed a higher likelihood of nutritional risk compared with the reference group (<70 years) (OR 1.53; 95% CI: 1.00–2.32; *p* = 0.04), whereas the group aged 70–79 years did not show statistically significant differences. Categorisation of the Charlson Comorbidity Index did not substantially modify the observed associations, confirming the robustness of the main model.

### 3.3. Clinical Consequences of Nutritional Risk

Patients at nutritional risk had a significantly longer hospital stay than those without nutritional risk, with a median length of stay of 10 days (IQR 11) versus 7 days (IQR 8) (*p* < 0.001). Nutritional risk was associated with a higher likelihood of prolonged hospital stay (defined as a stay longer than the 75th percentile, corresponding to 15 days) in the univariate analysis (OR 1.60; 95% CI: 1.23–2.09; *p* < 0.001). This association remained significant after adjustment in the multivariable model (OR 1.38; 95% CI: 1.01–1.88; *p* = 0.041) (Supplementary Table S4).

In-hospital mortality was higher in patients with nutritional risk compared with those without risk (11.7% vs. 6.2%; *p* < 0.001), with an unadjusted odds ratio of 2.02 (95% CI: 1.33–3.07). In the multivariable models, nutritional risk at admission was associated with higher 30-day mortality (adjusted OR 1.63; 95% CI: 1.12–2.37) and 60-day mortality after discharge (adjusted OR 1.53; 95% CI: 1.10–2.13). For in-hospital mortality, the magnitude of the association was similar, although it did not reach statistical significance after adjustment (OR 1.48; 95% CI: 0.88–2.50) (Supplementary Table S5).

**Figure 1:**
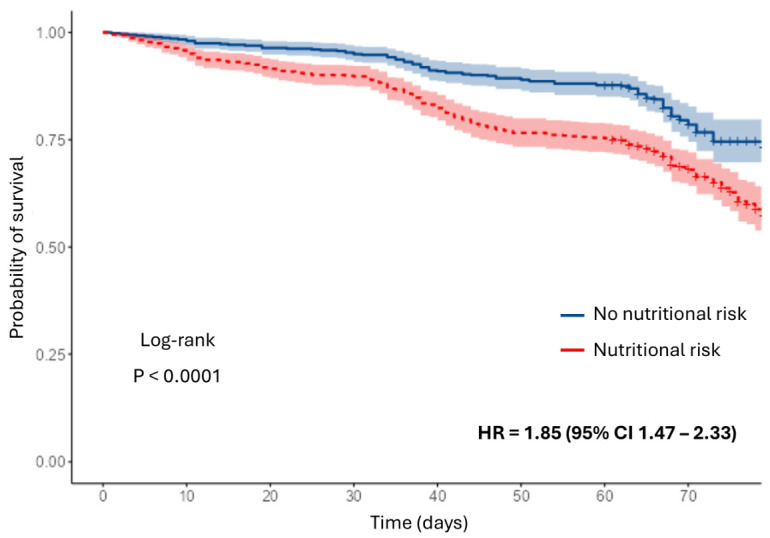
Overall survival according to the presence or absence of nutritional risk at hospital admission.

Kaplan–Meier survival analysis showed lower survival among patients with nutritional risk at admission compared with those without risk, with an early separation of the curves. This finding was consistent with the higher in-hospital mortality observed in patients at nutritional risk. The difference between groups was statistically significant according to the log-rank test (Figure 1).

No statistically significant differences were observed in the 60-day hospital readmission rate between patients with and without nutritional risk, either in the univariate analysis or after multivariable adjustment.

## 4. Discussion

This study represents the largest multicentre cohort conducted in Spain specifically focusing on nutritional assessment in hospitalised patients with cancer. In this population, 53% of patients were identified as being at nutritional risk according to the MST, highlighting the substantial burden of nutritional vulnerability among patients admitted to medical oncology services. In Spain, two major multicentre studies have previously evaluated the prevalence of hospital malnutrition: the PREDyCES study [26] (2012, screening using NRS-2002 in 31 centres) and the SeDREno study [17] (2021, 17 hospitals, assessment using MUST and GLIM criteria). However, neither of these studies was specifically designed to evaluate patients with cancer, who could also be hospitalised in any hospital department. A subanalysis of the PREDyCES study [16] including hospitalised oncology patients (N = 401) reported a prevalence of nutritional risk at admission of 33.9%, although only 15% of these patients were admitted to medical oncology departments. In the SeDREno study [17], 39% of patients with cancer (N = 466) presented nutritional risk, with confirmed malnutrition according to GLIM criteria in 39.1% of cases. In both studies, the prevalence of nutritional risk was substantially lower than that observed in our cohort. When focusing specifically on patients admitted to medical oncology departments, higher estimates have been reported. A recent single-centre study by López-Gómez JJ et al. [27] described a prevalence of nutritional risk of 67.8% using MUST and 91.3% using MNA, although the study included a relatively small sample size. International studies have also reported heterogeneous prevalences, including 39% malnutrition in France [18], 30.5% in Korea [28], 40.7% in the Netherlands [29] and 45.3% in Brazil [11]. Nevertheless, comparisons across studies should be interpreted with caution, as the prevalence of nutritional risk is influenced by several factors, including patient characteristics, tumour types, oncological treatments, reasons for admission, and the nutritional screening tools used.

In our study, nutritional risk was independently associated with functional status, tumour type, control of oncological disease, and recent exposure to cytotoxic chemotherapy. Rather than representing causal relationships, these factors likely reflect the coexistence of multiple markers of disease severity and patient vulnerability. Regarding tumour type, the highest prevalence of nutritional risk was observed in pancreatic cancer and tumours of the upper gastrointestinal tract, whereas breast cancer showed the lowest risk. These findings are consistent with previous reports describing a particularly high nutritional burden in malignancies affecting the digestive tract [11,14,16,18]. An unexpected observation was the high proportion of patients with prostate cancer classified as being at nutritional risk (58%), despite this malignancy generally being considered to carry a low nutritional risk profile. This finding likely reflects the specific clinical context of hospitalisation in medical oncology, which often includes patients with advanced disease, complications requiring admission, or exposure to intensive systemic treatments. One particularly relevant finding is that nutritional risk was not associated with tumour stage but rather with the level of disease control. Independent of metastatic status, patients at initial diagnosis or with progressive disease showed the highest prevalence of nutritional risk, whereas those with stable disease or objective oncological response presented substantially lower rates. Although this observation is clinically and pathophysiologically plausible, it contrasts with several previous studies reporting a direct association between malnutrition and tumour stage [30]. Age was not independently associated with nutritional risk when analysed as a continuous variable. However, when categorised, patients aged over 80 years showed a higher probability of nutritional risk, suggesting that the relationship between age and malnutrition may not be linear but instead concentrated at very advanced ages. This pattern is compatible with a threshold effect potentially linked to frailty or reduced physiological reserve, and similar findings were reported by Almeida et al. [31] in a cohort of more than 3000 older patients with cancer.

Our study confirms an independent association between exposure to cytotoxic chemotherapy and nutritional risk, consistent with previous reports [14,18]. This relationship is likely bidirectional, reflecting a vicious cycle in which malnutrition worsens treatment tolerance while treatment-related toxicity and complications further compromise nutritional status [7,21,30]. A more exploratory observation in our cohort relates to the potential relationship between immunotherapy and nutritional status, an area that remains poorly characterised in the current literature [14]. In our study, the prevalence of nutritional risk among patients receiving immunotherapy alone was slightly lower than that observed in patients treated exclusively with chemotherapy, but clearly higher than that observed in those receiving targeted molecular therapies or hormonal treatment. These findings should be interpreted with caution, as this analysis was not a predefined objective of the study, and the results should therefore be considered hypothesis-generating.

In this cohort of hospitalised patients with cancer, nutritional risk was consistently associated with worse clinical outcomes. Patients identified as being at nutritional risk experienced longer hospital stays and higher mortality, with a median hospitalisation three days longer and a significantly higher probability of prolonged admission (>15 days). These findings are in line with previous studies and recent meta-analysis [3,16,27,31,32] and highlight the clinical and economic implications of malnutrition in hospitalised oncology patients, particularly in terms of healthcare resource utilisation and associated costs [16]. While the association between nutritional status and survival has been widely described in ambulatory oncology populations [30,33,34,35], evidence in hospitalised patients remains comparatively scarce. In our study, patients at nutritional risk showed substantially higher mortality, both during hospitalisation and at 30 and 60 days after discharge, with mortality rates approximately twice those observed in patients without nutritional risk. Importantly, this association remained independent of other prognostic factors, as confirmed in the multivariable models. Although the association with in-hospital mortality did not reach statistical significance after adjustment, the consistent magnitude of the effect observed across the different time horizons (OR 1.4–1.6) supports the interpretation of nutritional risk at admission as a relevant prognostic marker in this population. The lack of statistical significance for in-hospital mortality likely reflects the lower number of events during hospitalisation and the resulting limited statistical power. Overall, our results are consistent with those reported in previous studies of hospitalised oncology patients, although most of these studies included smaller cohorts [36] and/or were conducted in single-centre settings [27,36,37,38].

In the general hospitalised population, the association between nutritional status and the risk of hospital readmission is well documented [39,40]. In contrast, evidence in patients with cancer remains more limited and heterogeneous [41,42]. In our cohort, no significant differences were observed in the 60-day readmission rate between patients with and without nutritional risk at admission. A possible explanation for this finding is the higher early mortality observed among patients with cancer, which may have reduced the likelihood of readmission being observed, with death acting as a competing risk.

This study has several strengths, including its prospective design, large sample size, and multicentre structure, with a representative distribution of the most common tumour types. Importantly, the study was specifically designed to characterise patients admitted to medical oncology departments, which distinguishes it from previous cohorts. However, several limitations should be acknowledged. First, nutritional status was assessed using MST, a screening tool for nutritional risk, and a comprehensive diagnostic evaluation of malnutrition based on GLIM criteria was not performed, despite these criteria currently being considered the diagnostic reference standard [2,17]. Therefore, our findings should be interpreted as referring to nutritional risk rather than confirmed malnutrition. However, the objective of the study was to evaluate nutritional screening at hospital admission in a large multicentre real-world cohort, and MST was selected because of its feasibility, simplicity, and prior validation in oncology settings. MST-based nutritional risk showed consistent associations with clinically relevant outcomes, supporting its usefulness as a pragmatic screening tool in hospitalised patients with cancer. In addition, sarcopenia was not evaluated, which may have limited a more complete characterisation of nutritional status. Furthermore, information on nutritional interventions during hospitalisation (e.g., oral nutritional supplements, enteral or parenteral nutrition) was not systematically collected. Nutritional management was performed according to routine clinical practice at each centre and was not standardised or protocol-driven within the study. Therefore, we were unable to evaluate the potential impact of nutritional support on clinical outcomes, although previous studies have suggested that nutritional interventions may improve outcomes, including mortality, in hospitalised patients with cancer [43]. Second, the generalisability of our findings to other clinical contexts may be limited, such as surgical oncology admissions or hospitalisations related to initial diagnostic work-up. Furthermore, in our healthcare system, patients with cancer receiving radiotherapy as their only treatment are rarely admitted to medical oncology departments, and therefore, this group may be underrepresented in our cohort. The REGIO registry was designed to assess multiple clinical dimensions of hospitalised oncology patients, which justified the use of a simple, rapid, and efficient nutritional screening tool. Moreover, most studies evaluating hospital malnutrition in oncology populations rely on screening tools rather than full diagnostic assessments, making our findings comparable with the existing literature. The choice of MST as the screening instrument may also have influenced our results, as different screening tools have shown partially non-overlapping diagnostic performance [21,22,32]. Nevertheless, MST is recommended by several major scientific societies for nutritional screening in oncology patients [7,8,10]. Finally, our findings suggest that MST provides adequate prognostic discrimination, as patients identified as being at nutritional risk showed worse clinical outcomes, supporting its practical usefulness in the hospital setting.

## 5. Conclusions

In this multicentre cohort of patients hospitalised in medical oncology departments, we observed a high prevalence of nutritional risk identified using MST, exceeding that previously reported in other national cohorts. Nutritional risk was independently associated with functional status (ECOG), tumour type, disease control, and recent exposure to cytotoxic chemotherapy. Importantly, patients identified as being at nutritional risk experienced worse clinical outcomes, including longer hospitalisation and higher mortality during follow-up, supporting the clinical relevance of systematic nutritional screening in hospitalised patients with cancer.

## Figures and Tables

**Table 1:** Demographic and clinical characteristics of patients in the REGIO cohort and stratification by nutritional risk (NR) defined by MST ≥ 2.

Baseline Characteristics	Total	No NR	NR	*p* Value *
Patients; n (%)	1229	568 (47)	641 (53)	
Age in years; median (IQR)	67.8 (15.75)	67.10 (15.2)	68.5 (15.97)	0.04
Male sex; n (%)	713 (59)	305 (53.8)	408 (63.7)	<0.001
Non-cancer Charlson Comorbidity Index; median (IQR)	1 (1.25)	1 (1)	1 (2)	0.58
Non-cancer Charlson 0–1; n (%)	869 (75)	412 (75.8)	457 (74.2)	
Non-cancer Charlson 2–3; n (%)	233 (20.1)	108 (19.88)	125 (20.3)	
Non-cancer Charlson ≥4; n (%)	57 (4.91)	23 (4.2)	34 (5.5)	
BMI, kg/m^2^; median (IQR)	24.6 (6.09)	26.2 (6.05)	23.6 (6.08)	<0.001
Low BMI **; n (%)	229 (19.4)	58 (10.5)	171 (27.3)	<0.001
Type of hospital; n (%)				0.11
<200 beds	85 (7.0)	34 (6)	51 (8)	
200–500 beds	378 (31.3)	177 (31.1)	201 (31.4)	
500–1000 beds	572 (47.3)	262 (46.1)	310 (48.4)	
>1000 beds	174 (14.4)	95 (16.7)	79 (12.3)	
Performance status				<0.001
ECOG 0–1	736 (61.4)	413 (73)	323 (51)	
ECOG 2	318 (26.5)	108 (19)	210 (33.2)	
ECOG 3–4	145 (12.1)	45 (8)	100 (15.8)	
Reason for admission; n (%)				0.13
Treatment-related toxicity	175 (14.5)	85 (15.0)	90 (14.1)	
Infection	415 (34.5)	216 (38.2)	199 (31.2)	
Thromboembolic event	49 (4.1)	22 (3.9)	27 (4.2)	
Other medical complications	440 (36.6)	192 (34)	248 (38.9)	
Post-surgical complications	10 (0.8)	5 (0.9)	5 (0.8)	
Supportive care and symptom control	114 (9.5)	46 (8.1)	68 (10.7)	
Reason for discharge; n (%)				<0.001
Death	110 (9.3)	35 (6.3)	75 (12)	
Hospital discharge	952 (80.4)	485 (86.9)	467 (74.6)	
Transfer to another service (non-palliative)	41 (3.5)	20 (3.6)	21 (3.4)	
Transfer to palliative care unit	81 (6.8)	18 (3.2)	63 (10.1)	
30-day readmission; n (%)	282 (27.1)	127 (24.9)	155 (29.2)	0.11
60-day readmission; n (%)	407 (33.7)	184 (32.4)	223 (34.8)	0.38
30-day mortality; n (%)	253 (20.9)	76 (13.4)	172 (26.8)	<0.001
60-day mortality; n (%)	339 (27.6)	109 (19.2)	225 (35.1)	<0.001
Length of hospital stay, days; median (IQR)	8 (10)	7 (8)	10 (11)	<0.001

* *p*-values were determined using the χ^2^ test for categorical variables and the Mann–Whitney U test for continuous variables. ** Low BMI (<22 in patients aged ≥ 70 years and <20 in those aged < 70 years). Percentages were determined based on the number of patients with available data for each variable.

**Table 2:** Prevalence of nutritional risk according to tumour type. Tumour types are presented in descending order according to prevalence; estimates from small sample sizes should be interpreted with caution.

Tumour Type	Prevalence of Nutritional Risk n/N (%)
Germ cell tumour	4/5 (80)
Oesophageal and gastroesophageal junction cancer	27/37 (73)
Pancreatic cancer	66/94 (70.2)
Renal cell carcinoma	22/32 (68.8)
Gastric cancer	35/52 (67.3)
Ovarian cancer	30/52 (60)
Cancer of unknown primary (CUP)	6/10 (60)
Prostate cancer	23/40 (57.5)
Head and neck cancer	30/53 (56.6)
Colorectal cancer	80/144 (55.6)
Bladder and urinary tract cancer	36/66 (54.5)
Biliary tract cancer	23/44 (52.2)
Small cell lung cancer (SCLC)	33/65 (50.8)
Non-small cell lung cancer (NSCLC)	146/288 (50.6)
Endometrial cancer	8/19 (42.1)
Melanoma	8/21 (38.1)
Breast cancer	38/114 (33.3)
Sarcoma	6/18 (33.3)
Central nervous system tumours	4/12 (33.3)
Other	10/36 (27.8)
Cervical cancer	2/8 (25)
Hepatocellular carcinoma	1/6 (16.7)

**Table 3:** Oncological characteristics of patients and stratification by nutritional risk (NR) defined by MST ≥ 2.

Oncological Characteristics	Total	No NR	NR	*p* Value *
Tumour type; n (%)	1216			<0.001
Non-small cell lung cancer (NSCLC)	285 (23.7)	139 (24.6)	146 (22.9)	
Small cell lung cancer (SCLC)	65 (5.3)	32 (5.7)	33 (5.2)	
Breast cancer	112 (9.3)	74 (13.09)	38 (6)	
Ovarian cancer	52 (4.3)	21 (3.7)	30 (4.7)	
Oesophageal and gastroesophageal junction cancer	37 (3)	10 (1.7)	27 (4.2)	
Gastric cancer	51 (4.2)	16 (2.8)	35 (5.5)	
Pancreatic cancer	93 (7.7)	27 (4.8)	66 (10.3)	
Colorectal cancer	143 (11.9)	63 (11.2)	80 (12.5)	
Biliary tract cancer	43 (3.6)	20 (3.5)	23 (3.6)	
Hepatocellular carcinoma	6 (0.5)	5 (0.9)	1 (0.2)	
Head and neck cancer	52 (4.3)	22 (3.9)	30 (4.7)	
Renal cell carcinoma	32 (2.6)	10 (1.8)	22 (3.5)	
Bladder and urinary tract cancer	66 (5.4)	30 (5.3)	36 (5.6)	
Prostate cancer	40 (3.3)	17 (3)	23 (3.6)	
Melanoma	21 (1.7)	13 (2.3)	8 (1.2)	
Central nervous system tumours	12 (1%)	8 (1.4)	4 (0.6)	
Sarcoma	18 (1.5)	12 (2.1)	6 (0.9)	
Germ cell tumour	5 (0.4)	1 (0.2)	4 (0.6)	
Cancer of unknown primary (CUP)	10 (0.8)	4 (0.7)	6 (0.9)	
Other	33 (2.9)	25 (4.4)	10 (1.6)	
Endometrial cancer	19 (1.5)	10 (1.8)	8 (1.2)	
Cervical cancer	8 (0.7)	6 (1)	2 (0.3)	
Nutritional impact according to tumour type; n (%) **				<0.001
Low	133 (12)	87 (17.2)	46 (7.6)	
Intermediate	743 (67)	342 (67.9)	401 (66.2)	
High	233 (21)	75 (14.9)	158 (26.1)	
Metastatic disease; n (%)	781 (64.9)	352 (62.7)	418 (65.6)	0.27
Oncological status at admission; n (%)				<0.001
De novo diagnosis	283 (23.9)	111 (20)	172 (27.3)	
Complete response	75 (6.3)	52 (9.4)	23 (3.7)	
Partial response	103 (8.7)	53 (9.5)	50 (8)	
Stable disease	230 (19.4)	130 (23.4)	100 (15.9)	
Progression	492 (41.6)	209 (37.6)	283 (45)	
Oncological treatment; n (%)				
Cytotoxic chemotherapy	722 (59.7)	316 (55.6)	406 (63.3)	0.006
Immunotherapy	248 (20.5)	123 (21.6)	125 (19.5)	0.35
Targeted molecular therapy	162 (13.4)	87 (15.3)	75 (11.7)	0.06
Hormonal therapy	40 (3.3)	22 (3.9)	18 (2.8)	0.3
Radiotherapy	110 (10.8)	51 (8.9)	59 (9.2)	0.83
Single-agent therapy; n (%)				<0.001
Chemotherapy alone	520 (72.8)	220 (66.5)	300 (78.3)	
Immunotherapy alone	108 (15.1)	54 (16.3)	54 (14)	
Targeted molecular therapy alone	68 (9.5)	45 (13.5)	23 (6)	
Hormonal therapy alone	18 (2.5)	12 (3.6)	6 (1.6)	

* *p*-values were determined using the χ^2^ test for categorical variables and the Mann–Whitney U test for continuous variables. ** Low impact: breast cancer/melanoma; intermediate impact: lung/ovary/endometrium/colon/biliary/urothelial/kidney/prostate cancers; high impact: oesophagus/stomach/pancreas/head and neck cancers. Percentages were determined based on the number of patients with available data for each variable.

**Table 4:** Univariable and multivariable analysis of factors associated with nutritional risk.

Univariable Analysis of Risk Factors Associated with Nutritional Risk			
	Odds ratio	95% confidence interval	*p* value
Age	1.01	1–1.02	0.053
Male sex	1.5	1.19–1.89	<0.001
ECOG performance status (ref: 0–1)			
2	2.49	1.89–3.27	<0.001
3–4	2.84	1.94–4.16	0.001
Non-cancer Charlson Index	1.06	0.97–1.16	0.222
Hospital > 500 beds	0.91	0.72–1.15	0.439
Progression vs. stable disease	1.93	1.51–2.46	<0.001
Metastatic disease	1.14	0.90–1.45	0.274
Tumour according to nutritional impact (ref: low)			
Intermediate	2.22	1.51–3.26	<0.001
High	3.95	2.54–6.25	<0.001
Cytotoxic chemotherapy	1.37	1.09–1.71	0.006
**Multivariable analysis of risk factors associated with nutritional risk (N = 1035)**			
	Odds ratio	95% confidence interval	*p* value
Age	1.01	0.99–1.02	0.11
Male sex	1.15	0.86–1.55	0.35
ECOG performance status (ref: 0–1)			
2	2.78	2.02–3.83	<0.001
3–4	2.97	1.91–4.62	<0.001
Non-cancer Charlson Index	0.998	0.89–1.11	0.96
Progression vs. stable disease	1.88	1.43–2.47	<0.001
Tumour according to nutritional impact * (ref: low)			
Intermediate	1.82	1.16–2.84	0.009
High	3.32	1.97–5.60	<0.001
Cytotoxic chemotherapy	1.41	1.07–1.84	0.015

* Tumours with low nutritional impact: breast/melanoma; intermediate impact: lung/ovary/endometrium/colon/biliary/urothelial/kidney/prostate; high impact: oesophagus/stomach/pancreas/head and neck.

## Data Availability

The original contributions presented in this study are included in the article/Supplementary Materials. Further inquiries can be directed to the corresponding author.

## Supplementary Materials

The following supporting information can be downloaded at https://www.mdpi.com/article/10.3390/nu18081296/s1, Table S1: Participating hospitals and hospital size, ordered by number of included patients; Table S2: Malnutrition Screening Tool; Table S3: Proportion of missing data across study variables; Table S4: Multivariable analysis of factors associated with prolonged hospital stay (≥15 days); Table S5: Multivariable analysis of factors associated with in-hospital mortality and mortality at 30 and 60 days after hospital discharge.

## Abbreviations

The following abbreviations are used in this manuscript:

AUC	Area under the curve
BMI	Body mass index
CI	Confidence interval
CUP	Cancer of unknown primary
DRM	Disease-related malnutrition
ECOG	Eastern Cooperative Oncology Group
GLIM	Global Leadership Initiative on Malnutrition
IQR	Interquartile range
MNA	Mini Nutritional Assessment
MST	Malnutrition Screening Tool
MUST	Malnutrition Universal Screening Tool
NR	Nutritional risk
NRS-2002	Nutritional Risk Screening 2002
NSCLC	Non-small cell lung cancer
OR	Odds ratio
SD	Standard deviation
SEMI	Spanish Society of Internal Medicine
SCLC	Small cell lung cancer
