# Supplementary material for: Nutritional Risk in Patients Admitted to Medical Oncology Departments: Prevalence, Associated Factors and Clinical Outcomes in a Multicentre Prospective Study
Source: Nutrients. 2026 Apr 20;18(8):1296. doi: 10.3390/nu18081296 (PMC13118729; doi:10.3390/nu18081296)
Supplement: Supplementary file 1 [file nutrients-18-01296-s001.zip › nutrients-4242686-supplementary.pdf]

Supplementary Table S1. Participating hospitals and hospital size, ordered by number of included patients.

Hospital	Hospital size (number of beds)	Included patients (%)
Hospital Parc Taulí, Sabadell	500-1000	182 (14.9)
Hospital Universitario 12 de Octubre	>1000	146 (11.9)
ICO Badalona (Hospital Universitari Germans Trias i Pujol)	500-1000	146 (11.9)
Hospital San Pedro, Logroño	500-1000	121 (9.9)
Hospital Clinic de Barcelona	500-1000	98 (8)
Hospital de Mataró	200-500	85 (6.9)
Hospital Virgen del Puerto, Plasencia	200-500	80 (6.5)
Hospital del Mar	500-1000	79 (6.4)
Hospital Universitario Arnau de Vilanova de Lleida	200-500	72 (5.9)
Hospital Universitario Infanta Cristina de Parla	<200	64 (5.2)
Hospital Universitario La Princesa	500-1000	35 (2.9)
Hospital Universitario Virgen de las Nieves, Granada	>1000	32 (2.6)
Hospital San Pedro de Alcántara, Cáceres	200-500	29 (2.4)
Complejo Hospitalario Ruber Juan Bravo	200-500	23 (1.9)
Hospital Quironsalud A Coruña	<200	21 (1.7)
Hospital Universitario Quironsalud Pozuelo	200-500	8 (0.7)
Hospital Nuestra Señora del Prado, Talavera de la Reina	200-500	4 (0.3)

Supplementary Table S2. Malnutrition Screening Tool

**Question**	**Score**
Have you lost weight recently without trying?	
No	0
Unsure	2
Yes	See below
If yes, how much weight (Kg) have you lost?	
1-5	1
6-10	2
11-15	3
>15	4
Unsure	2
Have you been eating poorly because of a decreased appetite?	
No	0
Yes	1
**Total score:**	Maximum 7
Interpretation:<br>0–1: Low risk of malnutrition<br>≥2: At risk of malnutrition	

Supplementary Table S3. Proportion of missing data across study variables

	Total	Missing	% missing

**Sex**	1228	1	0.08%
**Performance status (ECOG)**	1214	15	1.24%
**Non-tumor Charlson Comorbidity Index**	1172	57	4.86%
**Charlson Comorbidity Index**	1169	60	5.13%
**Age**	1207	22	1.82%
**Reason for admission**	1216	13	1.07%
**Oncological status at admission**	1197	32	2.67%
**Metastatic disease**	1215	14	1.15%
**Tumour type**	1216	13	1.07%
**Chemotherapy**	1229	0	0.00%
**Immunotherapy**	1229	0	0.00%
**Targeted molecular therapy**	1229	0	0.00%
**Hormonal therapy**	1229	0	0.00%
**Weight**	1211	18	1.49%
**Height**	1208	21	1.74%
**Body Mass Index**	1207	22	1.82%
**Malnutrition Screening Tool**	1209	20	1.65%
**Reason for discharge**	1197	32	2.67%
**Hospital stay**	1190	39	3.28%
**30-day readmission**	1055	174	16.49%
**30-day mortality**	1229	0	0.00%
**60-day mortality**	1229	0	0.00%
**Death during hospitalisation**	1229	0	0.00%
**Type of hospital**	1229	0	0.00%

Supplementary Table S4. Multivariable analysis of factors associated with prolonged hospital stay (≥15 days).

	OR (95% CI)	P value
**Nutritional risk (Yes vs No)**	**1.39 (1.02-1.89)**	**0.040**
ECOG		
3-4 vs 0-1	1.99 (1.28-3.09)	0.002
2 vs 0-1	1.78 (1.27-2.48)	<0.001
Non-tumor Charlson	0.99 (0.88-1.12)	0.867
Age (years)	0.99 (0.97-1.001)	0.067
Progression vs stable disease	1.29 (0.94-1.77)	0.117
Tumor according to nutritional impact		
Intermediate vs low nutritional impact	3.02 (1.70-5.41)	<0.001
High vs low nutritional impact	3.82 (2.01-7.28)	<0.001
Cytotoxic chemotherapy	0.72 (0.54-0.98)	0.034
Hospital size: (<500 vs >500 beds)	0.53 (0.39-0.72)	<0.001
Male sex	0.85 (0.62-1.16)	0.300

Tumors with low nutritional impact: breast / melanoma; Intermediate impact: lung / ovary / endometrium / colon / biliary / urothelial / kidney / prostate; High impact: oesophagus / stomach / pancreas / head and neck.

Supplementary Table S5. Multivariable analysis of factors associated with in-hospital mortality and mortality at 30 and 60 days after hospital discharge.

	During hospitalisation		30 days after discharge		60 days after discharge	
	OR (95% CI)	p value	OR (95% CI)	p value	OR (95% CI)	p value
**Nutritional risk (Yes vs No)**	**1.48 (0.88-2.50)**	**0.141**	**1.65 (1.13-2.41)**	**0.010**	**1.57 (1.13-2.19)**	**0.007**
ECOG						
3-4 vs 0-1	6.88 (3.83-12.35)	<0.001	7.92 (4.98-12.60)	<0.001	7.15 (4.58-11.17)	<0.001
2 vs 0-1	2.46 (1.41-4.30)	0.002	2.73 (1.85-4.04)	<0.001	2.77 (1.97-3.9)	<0.001
Progression vs stable disease	2.29 (1.25-4.22)	0.007	3.20 (2.05-4.99)	<0.001	2.40 (1.68-3.45)	<0.001
Metastatic disease	1.79 (1.01-3.17)	0.047	2.47 (1.62-3.77)	<0.001	1.89 (1.33-2.69)	<0.001
Male sex	0.65 (0.40-1.06)	0.085	0.82 (0.56-1.20)	0.306	0.93 (0.66-1.31)	0.688
Non-tumor Charlson	0.89	0.251	1.03	0.678	1.10	0.110

	(0.73-1.08)		(0.90-1.18)		(0.98-1.24)	
Age (years)	1.01 (0.99-1.032)	0.401	0.99 (0.98-1.02)	0.905	0.99 (0.98-1.004)	0.170
Tumor according to nutritional impact						
Intermediate vs low nutritional impact	2.49 (0.97-6.41)	0.058	2.42 (1.22-4.78)	0.011	2.09 (1.17-3.76)	0.013
High vs low nutritional impact	2.59 (0.91-7.39)	0.076	2.82 (1.32-6.05)	0.008	2.81 (1.46-5.42)	0.002

Tumors with low nutritional impact: breast / melanoma; Intermediate impact: lung / ovary / endometrium / colon / biliary / urothelial / kidney / prostate; High impact: oesophagus / stomach / pancreas / head and neck.
